# Population segmentation based on healthcare needs: a systematic review

**DOI:** 10.1186/s13643-019-1105-6

**Published:** 2019-08-13

**Authors:** Jia Loon Chong, Ka Keat Lim, David Bruce Matchar

**Affiliations:** Program in Health Services and Systems Research, Duke-NUS Medical School, Singapore, Singapore

**Keywords:** Population segmentation, Integrated care, Health care reform, Community health planning, Health services needs and demand, Person-focused health

## Abstract

**Background:**

Healthcare needs-based population segmentation is a promising approach for enabling the development and evaluation of integrated healthcare service models that meet healthcare needs. However, healthcare policymakers interested in understanding adult population healthcare needs may not be aware of suitable population segmentation tools available for use in the literature and barring better-known alternatives, may reinvent the wheel by creating and validating their own tools rather than adapting available tools in the literature. Therefore, we undertook a systematic review to identify all available tools which operationalize healthcare need-based population segmentation, to help inform policymakers developing population-level health service programmes.

**Methods:**

Using search terms reflecting concepts of population, healthcare need and segmentation, we systematically reviewed and included articles containing healthcare need-based adult population segmentation tools in PubMed, CINAHL and Web of Science databases. We included tools comprising mutually exclusive segments with prognostic value for clinically relevant outcomes. An updated secondary search on the PubMed database was also conducted as the last search was conducted 2 years ago. All identified tools were characterized in terms of segment formulation, segmentation base, whether they received peer-reviewed validation, requirement for comprehensive electronic medical records, proprietary status and number of segments.

**Results:**

A total of 16 unique tools were identified from systematically reviewing 9970 articles. Peer-reviewed validation studies were found for 9 of these tools.

**Discussion and conclusions:**

The underlying segmentation basis of most identified tools was found to be conceptually comparable to each other which suggests a broad recognition of archetypical patient overall healthcare need profiles. While many tools operate based on administrative record data, it is noted that healthcare systems without comprehensive electronic medical records would benefit from tools which segment populations through primary data collection. Future work could therefore include development and validation of such primary data collection-based tools. While this study is limited by exclusion of non-English literature, the identified and characterized tools will nonetheless facilitate efforts by policymakers to improve patient-centred care through development and evaluation of services tailored for specific populations segmented by these tools.

**Electronic supplementary material:**

The online version of this article (10.1186/s13643-019-1105-6) contains supplementary material, which is available to authorized users.

## Background

Given the worldwide trend of ageing populations and increasing prevalence of chronic disease [1], healthcare policymakers increasingly recognize the need to improve the management of patients with chronic diseases through patient-centred care [2, 3]. The integration of care centred on patient healthcare needs represents a paradigm shift which has been widely acknowledged to hold great potential for improving the quality of care [4]. However, given that no two patients are alike, the creation of models of care tailored for every individual in a population can become a prohibitively expensive and intractable endeavour [5]. Thus, segmenting populations into parsimonious groups that are relatively homogenous in terms of their healthcare needs helps in the design of integrated care models for the different population segments so as to optimally meet patients’ healthcare needs [5–7]. For example, Hewner et al. were able to demonstrate a reduction in hospitalization rates due to risk-stratified care management [7]. In this study, we define a healthcare need as a capacity to benefit from healthcare, where benefit can be quantified in various ways, depending on the outcomes of primary relevance to healthcare policymakers (e.g. cost-effectiveness, reduced disease incidence, decreased probability of transitioning into a worse health state, etc.) [8, 9].

Population segmentation is a concept that originated in the fields of business and marketing where product features are often tailored to meet the unique requirements of different market segments [10]. In a healthcare context, the benefits of population segmentation analysis for the provision of patient-centred care include the facilitation of healthcare needs evaluation, outcome tracking and care integration [11]. When a population is segmented into groups with similar patterns of healthcare need, policymakers may better understand a heterogenous population thus facilitating the planning of healthcare resources and interventions [5, 6]. An example of one such healthcare need-based scheme would be the ‘Bridges to Health’ model by Lynn et al. [6]. The Bridges to Health is seminal in proposing a population segmentation approach to population health and has been cited by a number of subsequent tools such as the Lombardy Segmentation scheme [12] and British Columbia Health System Matrix [13]. Tailoring services to needs is important as inadequate services lead to worse outcomes while excessive services lead to higher healthcare costs with little or no benefit to patients [14].

Population segmentation tools play an integral role in enabling the creation and refinement of integrated care systems [11]. While the potential value of population segmentation is appreciated, it is not self-evident how segmentation can be operationalized. Therefore, most health systems may choose to build their own tools barring better alternatives. This can cause evaluations comparing healthcare intervention effectiveness in different health systems to become more difficult as the different populations are segmented in a dissimilar manner.

Health systems are designed to meet healthcare needs. Four phenomena underlie the presence of need, namely risk of morbidity, pain/discomfort, dysfunction and risk of mortality [15]. In addition, need can be subjectively felt and expressed by patients themselves or normatively assessed by providers [9]. Currently, however, most population segmentation systems tend to be healthcare utilization risk-based [16] rather than healthcare needs-based. This is not ideal as healthcare need for specific healthcare services can occur independently of morbidity risk. Therefore, patient stratification based on morbidity risk, while useful as a proxy for intensity of need, is insufficient for informing the development of healthcare services in order to reduce this risk [17].

In contrast, segmenting patient populations based on healthcare needs provides indications for healthcare interventions that may reduce morbidity risk. For example, identifying patients as being in the healthcare need-based population category of ‘Limited reserve and serious exacerbations’ [6] informs us that priority concerns for this type of patient include exacerbation avoidance, with major components of healthcare comprising self-care support and 24/7 on-call access to medical guidance [6]. Healthcare needs-based segmentation thus has the distinct advantage of enabling development and evaluation of integrated services, in relation to the need for these services.

Although there have been reviews of general segmentation strategies with a focus on use-based schemes [18], to the best of our knowledge, no systematic review of healthcare need-based segmentation tools for adult populations has ever been conducted. Therefore, to bridge this gap in the literature and provide an overview of available population segmentation instruments for policymakers, we conducted a systematic review of all adult population healthcare need-based segmentation tools.

The primary objective of this study was to perform a systematic review of adult population healthcare needs-based segmentation tools. Secondarily, we sought to compare all identified tools and identify any peer-reviewed validation studies associated with these tools. Examples of validation include the demonstration of the prognostic ability of population segments for clinically relevant outcomes.

## Methodology

### Inclusion and exclusion criteria

Our inclusion criteria were as follows:

- Completely data-driven or non-completely data-driven population segmentation tools for overall healthcare needs. Data-driven tools are segmentation schemes that were typically generated ‘post-hoc’ using statistical clustering methods on a population dataset. Non-completely data-driven tools are schemes that were synthesized with at least some expert inputs on the segmentation criteria basis.
- Segmentation of the entire adult population whereupon an adult is defined as an individual who is 21 years old and above. May include schemes that account for study populations below age 21 provided adult populations are also segmented.
- Population segments offer possible prognostic value for clinically relevant outcomes (e.g. utilization, cost, morbidity. However, the segmentation basis cannot be solely prognostic outcome-based). Prognostic ability of segments was used as a proxy indicator of clinically meaningful, needs-based segmentation.
- Population segments are mutually exclusive. Thus, an individual can only exist in any one of the segments at any point in time.
- For identified tools with more than 10 referencing studies including validation studies, we only included studies with validation component of said tool.

Meanwhile, we excluded non-English literature and segmentation schemes that are based on single variables only such as frailty, physical function, utilization risk, health-related quality of life or single healthcare service need. The rationale for exclusion of such schemes is to optimize the segments to capture an overall healthcare need quality that will inform the development and evaluation of integrated packages of services for different population segments.

### Search and data extraction strategy

**Fig. 1:**
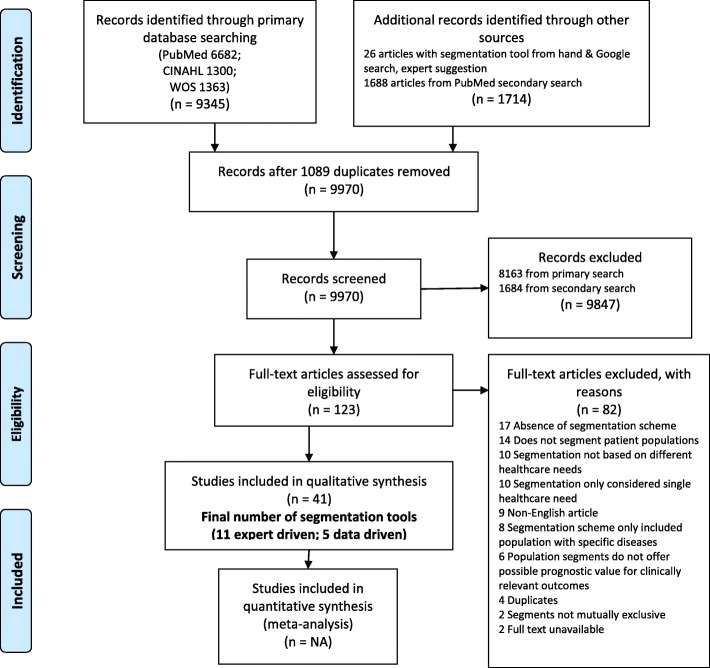
Systematic review workflow. CINAHL, Cumulative Index to Nursing and Allied Health Literature; WOS, Web of Science

Terms that were utilized revolved around the concepts of population, healthcare, segmentation and need. We considered synonyms, British/American phrasing and conceptually similar terms, as well as utilized quotation marks to improve the relevance of our hits. A search was planned for the 5th of January 2016 for all literature to-date in PubMed, CINAHL and Web of Science databases. (Detailed search terms, databases utilized can be found in Additional file 1: Appendix A, while PRISMA adherence can be viewed on Additional file 2: PRISMA Checklist, and Fig 1: PRISMA Flow Diagram/ Systematic review workflow).

After removing duplicates, all articles were screened in terms of title and abstract by 2 reviewers independently (JLC and KKL) and articles were shortlisted for full-text assessment independently by the same reviewers. All disagreements between reviewers were resolved through discussion. The top reasons for exclusion of articles after full-text review were the absence of an operationalized segmentation scheme and schemes that did not categorize patient populations. Eleven articles were shortlisted after full-text review.

Thereafter, hand searching was conducted independently on all 11 included articles by the same 2 reviewers (JLC and KKL). We also employed Google searches and consulted experts to identify possible segmentation schemes in the grey literature.

In addition to the primary systematic search detailed above, a secondary search was conducted on 7th July 2018. This search involved a review of the PubMed database using identical search terms as the primary search, as well as hand searching of previously included articles using Google scholar by the first author (JLC). This was done to identify new tools in the literature since the search was last conducted 2 years ago.

The total number of articles screened, including hand and Google search from both primary and secondary searches, was 9970 (Fig. 1). Data extraction of peer-reviewed validation studies was performed by both reviewers (JLC and KKL) independently (detailed data table in Additional file 1: Appendix D)

### Quality—risk of bias appraisal

All peer-reviewed validation studies were independently assessed by both reviewers (JLC and KKL) for quality using the QUIPS (Quality in Prognosis Studies) tool [19]. A consensus was then achieved through discussion of rating disagreements. While QUIPS was originally designed for evaluation of prognostic studies rather than segmentation scheme validation studies, we have adopted it for our analysis given that in this study, prognostic ability of segments was used as a proxy indicator of clinically meaningful, needs-based segmentation (Additional file 1: Appendix E). QUIPS assesses the risk of bias in the following 6 domains: study participation, study attrition, prognostic factor measurement, outcome measurement and study confounding, as well as statistical analysis and reporting. For this study, we deemed a study as overall ‘low risk of bias’ when it has a rating of ‘unlikely’ risk of bias across all 6 domains. Meanwhile, a study is deemed to have ‘high risk of bias’ when it has any ‘very likely’ risk of bias ratings across any of the 6 domains. All other studies were deemed to have ‘moderate risk of bias’.

### Data analysis: segmentation tool characterization

Upon completion of search, all tools were described in terms of their segment formulation, segmentation base type, peer-review validation status, proprietary status, need for comprehensive electronic medical records and number of segments (see Table 1). The method of tool creation or segment formulation describes the process by which the population segments were conceptualized. This can be done ‘a priori’ through expert inputs or ‘post-hoc’ using statistical methods in a data-driven manner (often followed by an interpretive step). Segmentation base type refers to the information type required by segmentation logic to assign individuals to segments [20]. Information types include demographic (e.g. age, gender, etc.), medical (e.g. diagnosis, prescribed medications, cognitive ability, etc.) and social (e.g. lifestyle, loneliness, functional status, etc.). Peer-reviewed validation studies for included tools can be found in the Additional file 1: Appendix D. Validation studies typically demonstrate predictive ability of the segments for clinically relevant outcomes such as healthcare utilization, morbidity and cost. A tool is said to be proprietary if it is used under exclusive legal right of the inventor or owner, while a comprehensive electronic medical record is defined as a record system which has sufficient scope (includes inpatient and outpatient records) as well as coverage (includes all individuals in a population of interest). Tools that do not require a comprehensive electronic medical record typically involve prospective data gathering.

**Table 1:** Characteristics of identified tools

Segmentation tool	Segment formulation	Segmentation base type	Peer-reviewed validation	Proprietary	Need for comprehensive electronic medical record	Number of segments
Lynn et al.’s Bridges to Health model	Expert driven	Medical	No	No	No	8
Hewner et al.’s Complexedex	Expert driven	Medical, lifestyle	No	Yes	Yes	4
Kaiser Permanente’s Senior Segmentation Algorithm (SSA)	Expert driven	Medical	Yes	Yes	Yes	4
Delaware Population Grouping	Expert driven	Medical	No	No	Yes	20
Lombardy Segmentation	Expert driven	Medical, demographic, utilization	No	No	Yes	8
3M’s Clinical Risk Group (CRG)	Expert driven	Medical, demographic	Yes	Yes	Yes	6–269
Joynt et al.’s Medicare claims-based segmentation	Expert driven	Medical, frailty indicators, demographic	Yes	No	Yes	6
British Columbia Health System Matrix	Expert driven	Medical, demographic, utilization	No	No	Yes	14
Singapore MOH (Ministry of Health) Segmentation framework	Expert driven	Medical, utilization	Yes	No	Yes	6
Northwest London Segmentation Scheme	Data, expert driven	Medical, demographic, functional	No	No	Yes	10
John Hopkins Adjusted Clinical Group (ACG)	Data, expert driven	Medical, demographic	Yes	Yes	Yes	92
Van der Laan et al.’s Demand-driven segmentation model	Data driven	Medical, functional, social	Yes	No	No	5
Liu et al.’s Latent Class Analysis (LCA) of Taiwan National Health Interview Survey (NHIS)	Data driven	Medical, functional, socio-demographic	Yes	No	No	4
Lafortune et al.’s LCA of SIPA (French acronym for System of Integrated Care for the frail elderly) Trial	Data driven	Medical, functional, socio-demographic	Yes	No	No	4
Vuik et al.’s utilization-based segmentation	Data driven	Utilization	No	No	Yes	8
Low et al.’s utilization-based segmentation	Data driven	Utilization, demographic	Yes	No	Yes	5

### Data analysis: segment theme comparison

Next, we attempted to compare all identified tools conceptually based on their underlying segment themes (see Additional file 1: Appendix C). Population segment concepts deemed by the authors to be conceptually similar were grouped together under a unifying segment theme. Thereafter, comparisons were made between tools in terms of the number of segment themes included by the respective schemes.

### Data analysis: segmentation tool characteristic based groupings

**Fig. 2:**
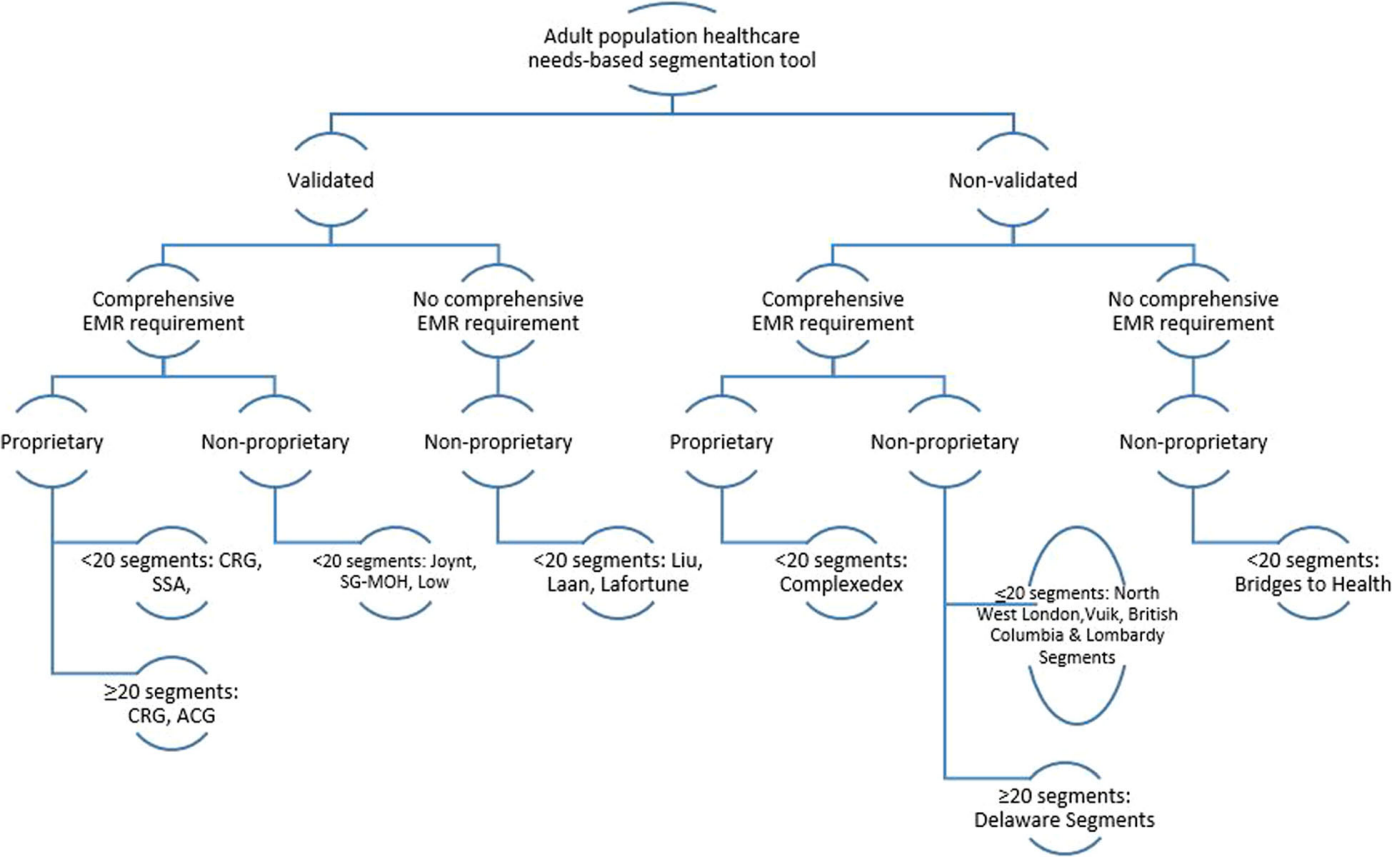
Categorization tree of identified segmentation tools. ACG, Adjusted Clinical Groups; CRG, Clinical Risk Groups; SSA, Senior Segmentation Algorithm; SG-MOH, Singapore Ministry of Health Segmentation Framework

Finally, we proposed a categorization tree to characterize the different types of segmentation tools (see Fig. 2). The intention of this exercise was to assist policymakers in choosing the tool which is most suitable for their respective healthcare systems. The selected variables for grouping tools were validation status, need for comprehensive electronic medical records, proprietary status and number of segments.

Validation status is relevant for policymaker consideration as non-validated tools should preferably be validated before they can be utilized to generate data that will inform public policy. Need for comprehensive electronic medical records is relevant to note as not all healthcare systems maintain a comprehensive electronic medical record. For example, in many countries, ICD diagnostic codes [21] may not be available in both inpatient and outpatient records, or patients may visit multiple healthcare institutions but policymakers have no access to the electronic medical records of all institutions. Thus, they will only be able to evaluate a patient partially using such tools. The quality and completeness of electronic medical record data can be quite variable too as evidenced by several studies which found that electronic medical record derived codes varied significantly from other validated data sources [22–26]. Nonetheless, for healthcare systems with a robust and comprehensive electronic medical record for their populations, these tools are potentially suitable and can lead to increased ease of identifying subjects within different population segments. Tool proprietary status is important to consider as such tools can be potentially expensive to licence, and users are not able to freely modify the tool’s internal logic to adapt for use in local contexts.

## Results

Our primary systematic search identified a total of 12 unique adult population healthcare need-based segmentation tools. Meanwhile, our secondary search identified a further 4 unique tools (detailed breakdown in Additional file 1: Appendix B). Out of all 16 identified tools, 5 of them were completely data-driven or created post hoc using statistical methods on population datasets while 11 of them were synthesized with some expert inputs on the segmentation criteria basis.

Table 1 illustrates the differences between all identified tools in terms of several selected variables which may impact the operationalization of a tool in different health systems and populations. The number of segments for some tools exist within a range as the segments are collapsible into larger or smaller segments depending on the desired level of granularity. Between tools that were expert driven and tools that were data driven, we note that data-driven tools utilized more segmentation bases that were non-medical, tended to be non-proprietary, as well as comprised typically of a smaller number of segments compared to expert-driven tools. Meanwhile as can be seen in Additional file 1: Appendix D, segmentation tool validation studies were found to be conducted in North America (e.g. USA, Canada), Europe (e.g. Spain, Sweden, Netherlands) and Asia (e.g. Singapore, Taiwan). Except for Adjusted Clinical Groups (ACG) and Clinical Risk Groups (CRG) which has been tested in both North American and European populations, all other tools are only known to have been utilized in its country of origin. The segmentation tool validation studies were further evaluated using an adapted QUIPS tool for risk of bias; ratings for individual studies are available in Additional file 1: Appendix F. Out of a total of 29 validation studies, 17 had overall ‘low risk of bias’, 8 had overall ‘moderate risk of bias’, and 4 had overall ‘high risk of bias’. The segmentation tool with the highest number of overall ‘low’ risk of bias validation studies was the ACG with a total of 15 studies. All other ‘low’ risk of bias validation studies evaluated the CRG (see Additional file 1: Appendix D). A sensitivity analysis which considered the rating of ‘maybe biased’ ratings for at least 2 QUIPS domains as an additional criteria for overall ‘high risk of bias’ only led to a change in overall risk of bias for Lafortune et al.’s study [27], which changed from overall ‘moderate’ to ‘high’ risk of bias (see Additional file 1: Appendix F).

A total of 11 segment themes were identified for all segmentation tools (See Additional file 1: Appendix C). They include: healthy, acute illness, maternal and infant health, minor chronic disease, moderate chronic disease, frailty, major chronic illness, cancer, mental illness, end-of-life and excluded subjects. These themes represent population segments with diverse and distinctive healthcare needs that exist at a population level. Therefore, schemes that include a larger number of these segment themes are potentially more actionable and responsive to tailored interventions. While none of the identified tools comprise all 11 segment themes, we note that tools with the highest number of segment themes were the British Columbia Health System Matrix (10 segment themes) and the Bridges to Health segmentation scheme (8 segment themes).

Next, we propose the following categorization tree to aid policymakers in understanding the different types of segmentation tools for purposes of adapting an instrument that best suits their healthcare systems’ needs (see Fig. 2).

### Validated proprietary tools

Validated tools that require comprehensive electronic medical records (EMR) can be divided into two groups based on their proprietary status. The proprietary group of tools include the Johns Hopkins Adjusted Clinical Groups (ACG) system [28–39], the 3M’s Clinical Risk Groups (CRG) [40–44] as well as Kaiser Permanente’s Senior Segmentation Algorithm (SSA) [45].

The Johns Hopkins ACG System utilizes diagnostic codes as well as demographic information such as age and gender as input data. Thereafter, using an algorithm, patients are classified into mutually exclusive Adjusted Clinical Groups [34]. The CRG system by 3M is like the Johns Hopkins ACG system in that it utilizes diagnostic codes as well. Patients are segmented into a mutually exclusive category based on a hierarchical system of classification where greater weightage is given to patients’ highest morbidity diseases [40]. Both segmentation systems have been validated through demonstration that patients categorized in different segments have differing expected healthcare utilization associated costs (see Additional file 1: Appendix D). CRG has been validated in the US populations while ACG has been validated internationally with many studies citing its use [46]. That said, both ACG and CRG create a large number of segments which may in turn make it difficult for policymakers to develop programmes to cater to every unique segment.

### Validated non-proprietary tools

All these tools were considered to have been validated at least internally as their respective authors were able to demonstrate predictive validity of the various segments. Validated, non-proprietary tools can in turn be divided into 2 groups based on whether these tools require a comprehensive electronic medical record.

### Validated non-proprietary tools: comprehensive EMR required

The tools that require a comprehensive electronic medical record were Joynt et al.’s Medicare claims-based segmentation [47], Low et al.’s utilization-based segmentation [48], and the Singapore MOH (Ministry of Health) Segmentation framework [49]. These tools sourced for medical, demographic and frailty indicator variables from administrative records to describe and classify their respective patient populations. Joynt et al. demonstrated that individuals in different segments have different risk of being high-cost patients while the Singapore MOH Segmentation framework was shown to be able to distinguish patient healthcare utilization using one-way ANOVA tests. Finally, Low et al.’s utilization-based segmentation scheme was able to segment patients into groups with different prospective healthcare utilization rates.

### Validated non-proprietary tool: comprehensive EMR not required

The tools which do not require a comprehensive electronic medical record were 3 post hoc segmentation schemes generated using latent class analysis on patient datasets which included both medical, functional and social data points (e.g. demand-driven segmentation model, latent class analysis (LCA) of the Taiwan NHIS, LCA of SIPA Trial). All 3 tools utilized self-reported disease status or unmet needs which included biological, psychological and functional domains. Van der Laan et al.’s study (demand-driven segmentation model) also examined social domain inputs [50]. Liu et al.’s (LCA of Taiwan NHIS) [51, 52] and Lafortune’s studies (LCA of SIPA trial) demonstrated using logistic regression the predictive ability of the segments for healthcare service utilization. Van der Laan et al.’s study on the other hand demonstrated descriptively that the segmentation results can be replicated in different populations within the Netherlands.

Policymakers who intend to utilize these schemes may need to instead perform primary data collection to determine relevant patient characteristics which allow them to be segmented into one of these patient categories. The strength of these tools is that they may utilize both medical and social healthcare needs as part of their patient data input logic which will result in a more holistic healthcare need profile. However, a limitation is that segmentation patterns vary for different populations. Thus, the generalizability of these tools merits further testing. There is also subjectivity when users of the tool attempt to synthesize clinical criteria which correlates with segments created through statistical technique (e.g. latent class analysis).

### Non-validated tools: comprehensive EMR required

Next, non-validated tools can be divided into those that require a comprehensive electronic medical record and those that do not. Tools that utilize a comprehensive electronic medical record include Vuik et al.’s utilization based segmentation scheme [5] and the segmentation schemes developed in Lombardy [12], North West London [17], British Columbia [13] and Delaware [53], as well as Complexedex [7, 17]. Complexedex is the only proprietary tool in this category. Most tools utilized demographic information such as age and disease condition information based on diagnostic codes as inputs. Complexedex also utilizes lifestyle data as its segmentation base while North West London segmentation also considers functional ability. Utilization pattern is used as a segmentation base for Vuik et al.’s, Lombardy and British Columbia segmentation schemes. All tools in this category are not considered to be validated as there were no peer-reviewed studies found which described the predictive ability of the respective schemes.

### Non-validated tools: comprehensive EMR not required

Finally, for non-validated tools that are not dependent on a comprehensive electronic medical record, there is only ‘Bridges to Health’ [6]. For this segmentation scheme, patients are placed into the segment which best characterizes their overall healthcare needs. While it is based on a relatively subjective assessment of medical- and health-related social needs, it nonetheless enjoys strong face validity among clinicians with regard to its ability to characterize the various typical types of patients that exist in any population and has been used widely [7, 45, 50]. This tool is suitable for consideration by policymakers who prefer to perform primary data collection to circumvent the limitations of a non-comprehensive electronic medical record. Notably, there is no formal primary data collection tool available to operationalize the Bridges to Health categories.

## Discussion

Historically, there is a consensus that the effectiveness of market segmentation strategy is determined by six criteria: *identifiability, substantiality, accessibility, stability, responsiveness* and *actionability* [20]. In the context of healthcare policy, an ideal population segmentation scheme for example, should thus be parsimonious in terms of number of segments so that each segment represents a relatively *substantial* portion of the population, as well as be *responsive* and *actionable,* such that each population segment is able to uniquely benefit in *response* to healthcare service interventions. To illustrate the qualities of *actionability, substantiality* and *identifiability* of some of the tools identified in this study, we have attempted to compare them based on their technical characteristics (see Table 1, Fig. 2) and underlying segment themes (see Additional file 1: Appendix C). Meanwhile, characterization of *accessibility, stability* and *responsiveness* of identified population segments are beyond the scope of the present study and will be part of future work. In determining the suitability of identified tools for specific healthcare systems, policymakers may consider both the characteristics of identified tools and the characteristics or priorities of their respective healthcare systems. Finally, with regards to the risk of bias appraisal of segmentation tool validation studies based on the adapted QUIPS framework, while most studies were deemed to have overall ‘low risk of bias’, those that were not were noted to have issues mainly with the QUIPS domains of study confounding and statistical analysis.

## Implications

The fact that many of the identified tools in this study are created to be used with a comprehensive electronic medical record suggests a strong dependence of effective population segmentation on a well-designed electronic medical record system. Ideally, the electronic medical record should be a platform which enables triangulation of patient healthcare need variables from various sources (e.g. hospital records, clinic records, patient-reported variables, etc.) to enable identification of a patient’s population segment. That said, because the quality of an electronic medical record system depends on inputs primarily from clinicians, there is a concomitant need to minimize the need to gather patient information which are not actionable to avoid unduly burdening clinicians. As things currently stand, the typical clinician may already be spending a significant amount of time daily on electronic medical record data entry [54]. By utilizing healthcare need-based tools such as the ones identified in this study, policymakers can tailor data entry fields on the electronic medical record based on variables required by the tool which has the dual benefit of enabling population healthcare need assessment while reducing clinician workload.

If required healthcare need variables are not found in the electronic medical record, an alternative is to conduct primary data collection using healthcare need assessment tools such as Easycare [55] and Interrai [56]. While these tools are useful for individual level (i.e. ‘micro’ level) care planning (which may take too much time to collect in the routine outpatient clinical setting), the patient variables required for population-level healthcare need assessment through segmentation typically does not require as many data elements. Therefore, one way to reduce the number of data elements needed would be to determine patient population features at the ‘meso’-level. Analysis at the meso-level is commonly used in the social sciences to point to a specific size and scale of research target which typically lies between ‘micro’ and ‘macro’ levels [57]. An example of ‘micro’ level assessment could be an individual’s specific activity of daily living ability in terms of ability to bathe oneself. However, when assessing a population, one may opt for meso-level assessment instead such as whether individuals have any impairment in their ability to conduct activities of daily living (i.e. yes/no). This enables formation of population segments based on common individual features which may have been assessed through different modalities at the individual level. As demonstrated in this study, most population segmentation base variables exist on the meso-level. Due to the summative characteristic of meso-level variables, primary data collection of such variables can potentially be less time consuming as well. That said, meso-level variables can be limited by a lack of specific features needed for tailoring services at an individual level thus cannot completely substitute for micro-level variables. In a nutshell, development and evaluation of primary data collection tools which collect meso-level healthcare need variables represent a possible area for future work.

Healthcare need-based population segmentation is an integral component of the epidemiological approach to population healthcare needs assessment as understanding of needs in relation to available services and their effectiveness promotes policymaker resource redistribution to optimize population health [58–60]. Currently, the outcomes of healthcare interventions are commonly evaluated through objective variables such as incidence of hospital admission and disease biomarkers [4]. By segmenting a population based on healthcare needs, healthcare interventions can be designed for patients within low-need population segments and the outcomes evaluated based on their rate of progression to higher needs population segments. This thus allows for the evaluation of health-related social services and preventive-type healthcare services. In addition, this also provides a new means of evaluating healthcare intervention ability to meet healthcare needs (i.e. effectiveness) for specific population segments by quantifying effectiveness as a reduction in the probability of progression to higher morbidity population segments because of specific healthcare interventions delivered.

## Strengths and limitations

In this systematic review, we identified 16 unique adult population healthcare need-based segmentation tools and proposed a framework to assist policymakers in choosing the most optimal tool for use in their respective healthcare systems. The strengths of this study include its novelty as the first systematic review for adult population healthcare need-based segmentation tools to our knowledge where both peer-reviewed and grey literature was reviewed. The literature evidence of predictive validity supporting the identified tools was also compiled and the tools themselves compared conceptually as well as operationally. Nonetheless, a limitation of this study is the exclusion of non-English literature and paediatric populations. Next, while we attempted to evaluate the risk of bias of segmentation tool validation studies using an adapted QUIPS framework, we acknowledge that QUIPS was designed mainly for prognosis studies which tend to be longitudinal cohort studies utilizing continuous variable-type prognostic factors. Finally, due to insufficient data for a quantitative meta-analysis, the heterogeneity of segmentation tools’ healthcare need covariates, as well as the lack of consensus on a framework for assessing the quality of healthcare need-based population segmentation tools, the results of this review are presented as a narrative summary.

## Conclusions

Population segmentation holds enormous potential to catalyse patient-centred care, yet healthcare need-based population segmentation remains a relatively novel approach with only a relatively limited number of validated tools available in the literature compared to healthcare utilization risk-based segmentation tools [16]. To further advance the field of healthcare need-based segmentation, future work could include further development and validation of currently identified non-validated tools for use in healthcare systems without comprehensive electronic medical records. This would likely involve the development of primary data collection-based tools as well if required segmentation base variables (e.g. health-related social care need variables) are not routinely collected by a healthcare system. In addition, studies that compare interchangeability of segments between hypothetically mappable tools may aid inter-population healthcare need profile comparisons. Finally, development and evaluation of service packages tailored to specific healthcare need-based population segments’ need enables a virtuous cycle of continual refinement of service interventions designed in relation to need. In conclusion, this study has found that only a limited number of healthcare need-based segmentation tools exist in the literature. This is an area that deserves greater attention as by planning health services through focusing on healthcare needs based population segments, policymakers may become better able to match healthcare services to needs, thus improving whole population health outcomes.

### Additional files

Additional file 1: **Appendix A**; detailed search terms. **Appendix B**; number of articles per tool. **Appendix C**; comparison of segment concepts. **Appendix D**; extracted segmentation tool validation data. **Appendix E**; adaptation of QUIPS tool for evaluation of segmentation tool validation studies. **Appendix F**; detailed QUIPS rating (DOCX 122 kb)

Additional file 2: PRISMA checklist (DOC 64 kb)

## Data Availability

The datasets generated and/or analysed during the current study are available from the corresponding author on reasonable request.

## Abbreviations

ACG: Adjusted Clinical Groups
CINAHL: Cumulative Index to Nursing and Allied Health Literature
CRG: Clinical Risk Groups
EMR: Electronic medical records
ICD: International Classification of Diseases
LCA: Latent class analysis
NHIS: National Health Interview Survey
SIPA: French acronym for System of Integrated Care for the frail elderly
SSA: Senior Segmentation Algorithm
