# Supplementary material for: Population segmentation based on healthcare needs: a systematic review
Source: Syst Rev. 2019 Aug 13;8:202. doi: 10.1186/s13643-019-1105-6 (PMC6693177; doi:10.1186/s13643-019-1105-6)
Supplement: Supplementary file 1 — Appendix A; detailed search terms. Appendix B; number of articles per tool. Appendix C; comparison of segment concepts. Appendix D; extracted segmentation tool validation data. Appendix E; adaptation of QUIPS tool for evaluation of segmentation tool validation studies. Appendix F; detailed QUIPS rating (DOCX 122 kb) [file 13643_2019_1105_MOESM1_ESM.docx]

# **Appendix A**

## **Search Terms**

| **Concepts** | **Synonyms** | **Search Terms** |
| --- | --- | --- |
| **population** | community | communit* |
|  | population | population* |
| **healthcare need** | health | "health need*" |
|  | healthcare | "health service* need*" |
|  | health service | "healthcare need*" |
|  | healthcare service | "healthcare service* need*" |
|  | health care | "health care need*" |
|  | health care service | "health care service* need*" |
|  | medical care | "medical care need*" |
|  | medical service | "medical service* need*" |
|  | biopsychosocial | "biopsychosocial need*" |
|  | bio-psychosocial | "bio-psychosocial need*" |
|  |  |  |
|  | need | "health requirement*" |
|  | requirement | "health service* requirement*" |
|  |  | "healthcare requirement*" |
|  |  | "healthcare service* requirement*" |
|  |  | "health care requirement*" |
|  |  | "health care service* requirement*" |
|  |  | "medical care requirement*" |
|  |  | "medical service* requirement*" |
|  |  | "biopsychosocial requirement*" |
|  |  | "bio-psychosocial requirement*" |
|  |  |  |
|  | utilisation | "healthcare utilisation" |
|  | utilization | "health service utilisation" |
|  |  | "healthcare service utilisation" |
|  |  | "health care utilisation" |
|  |  | "health care service utilisation" |
|  |  | "medical care utilisation" |
|  |  | "medical service utilisation" |
|  |  |  |
|  |  | "healthcare utilization" |
|  |  | "health service utilization" |
|  |  | "healthcare service utilization" |
|  |  | "health care utilization" |
|  |  | "health care service utilization" |
|  |  | "medical care utilization" |
|  |  | "medical service utilization" |
|  |  |  |
|  | risk | "health risk*" |
|  | status | "health status*" |
|  | profile | "health profile*" |
|  |  |  |
|  |  | "biopsychosocial risk*" |
|  |  | "biopsychosocial status*" |
|  |  | "biopsychosocial profile*" |
|  |  |  |
|  |  | "bio-psychosocial risk*" |
|  |  | "bio-psychosocial status*" |
|  |  | "bio-psychosocial profile*" |
| **segmentation** | type | typolog* |
|  | stratification | stratification* |
|  | segmentation | segmentation* |
|  | classification | classification* |
|  | categorization | categorization* |
|  | categorisation | categorisation* |

**Search term sample inputs**

((communit* OR population*)

AND

("health need*" OR "health service* need*" OR "healthcare need*" OR "healthcare service* need*" OR "health care need*" OR "health care service* need*" OR "medical care need*" OR "medical service* need*" OR "biopsychosocial need*" OR "bio-psychosocial need*" OR "health requirement*" OR "health service* requirement*" OR "healthcare requirement*" OR "healthcare service* requirement*" OR "health care requirement*" OR "health care service* requirement*" OR "medical care requirement*" OR "medical service* requirement*" OR "biopsychosocial requirement*" OR "bio-psychosocial requirement*" OR "healthcare utilisation" OR "health service utilisation" OR "healthcare service utilisation" OR "health care utilisation" OR "health care service utilisation" OR "medical care utilisation" OR "medical service utilisation" OR "healthcare utilization" OR "health service utilization" OR "healthcare service utilization" OR "health care utilization" OR "health care service utilization" OR "medical care utilization" OR "medical service utilization" OR "health risk*" OR "health status*" OR "health profile*" OR "biopsychosocial risk*" OR "biopsychosocial status*" OR "biopsychosocial profile*" OR "bio-psychosocial risk*" OR "bio-psychosocial status*" OR "bio-psychosocial profile*")

AND

(typolog* OR stratification* OR segmentation* OR classification* OR categorization* OR categorisation*))

## **Search results**

| Databases searched on 5th January 2016 | Hits |
| --- | --- |
| Pubmed | 6,682 |
| Web of Science (topic search) (all database) | 1,363 |
| CINAHL | 1,300 |
| Total | **9,345** |

| Updated search conducted on 6th July 2018 | Hits |
| --- | --- |
| Pubmed (updated search without counting previous 6682 hits) | 1,688 |

**Google Search Strategy**

Our google search strategy involved screening the title and abstract for articles with the search terms “population healthcare need segmentation” through the following specific approaches for all articles or at least 100 articles per approach, whichever is smaller in number, for articles dated up to the search date of 5^th^ January 2016. The searched journals were selected as segmentation tools were found from studies published in them from the systematic search. Likewise, the searched authors were selected as segmentation tools were found from studies published by them in the systematic search.

1. General Google Search
2. Google Scholar Search:
   1. General Google Scholar Search
   2. Journal Specific Google Scholar Search
      1. Milbank Quarterly
      2. Health Policy
      3. Population Health Management
      4. Health Services Research
      5. Medical Care
      6. BMC Health Services Research
      7. British Medical Journal
      8. JAMA
      9. International Journal of Integrated Care
   3. Author Specific Google Scholar Search
      1. Sabine Vuik
      2. Barbara Starfield
      3. M.R. Eissens van der Laan
      4. Liu Li-Fan
      5. Louise Lafortune
      6. Yi Yvonne Zhou
      7. Joanne Lynn
      8. Sharon Hewner

# **Appendix B**

| S/N | Tool | Number of articles | Source |
| --- | --- | --- | --- |
| 1 | Adjusted Clinical Groups | 17 | Systematic Review |
| 2 | Clinical Risk Groups | 6 | Systematic Review |
| 3 | Senior Segmentation Algorithm | 1 | Systematic Review |
| 4 | Bridges to Health | 1 | Systematic Review |
| 5 | LCA1 - Demand driven care | 1 | Systematic Review |
| 6 | LCA2 – SIPA Trial | 2 | Systematic Review |
| 7 | LCA3 - Taiwan National Health Insurance | 2 | Hand searching |
| 8 | Complexedex | 3 | Hand searching |
| 9 | Lombardy Segmentation | 1 | Hand searching |
| 10 | North West London Segmentation | 1 | Google searching |
| 11 | Delaware Segmentation | 1 | Google searching |
| 12 | British Columbia Health System Matrix | 1 | Expert suggestion |
| 13 | Joynt et al’s Medicare claims based segmentation | 1 | Secondary search |
| 14 | Singapore MOH Segmentation framework | 1 | Secondary search |
| 15 | Vuik et al’s utilization based segmentation | 1 | Secondary search |
| 16 | Low et al’s utilization based segmentation | 1 | Secondary search |
|  | **Total** | **41** |  |

Comment: This table corresponds to number of times a segmentation tool was found in a unique article out of all articles included in the review (N = 37, number of hits for segmentation tool). Source refers to the method through which the segmentation tool was first encountered. There is a higher number tool hit count than total number of articles reviewed (37 vs 36) as a single article can detail more than one segmentation tool. Meanwhile, appendix C only details segmentation tools with a validation component (N = 27, number of studies). Only 26 articles are included in Appendix C as one article detailed validation of 2 segmentation tools (1).

# **Appendix C**

| **Concept/ Segmentation Schemes** | **Delaware Population Grouping** | **British Columbia Health System Matrix** | **Northwest London Segmentation Scheme** | **Clinical Risk Group** | **Bridges to Health** | **Lombardy Segmentation Scheme** | **Vuik et al's utilization based segmentation** | **Joynt et al's Medicare claims based segmentation** | **Singapore MOH Segmentation framework** | **Low et al's utilization based segmentation** | **Demand-driven Care Segmentation Model** | **LCA of Taiwan NHIS** | **LCA of SIPA Trial** | **Complexedex** | **Senior Segmentation Algorithm** |
| --- | --- | --- | --- | --- | --- | --- | --- | --- | --- | --- | --- | --- | --- | --- | --- |
| **Healthy** | **Infants with No chronic conditions** | **Healthy/ minor episodic health needs** | **Mostly healthy adults <75 years old** | **Healthy** | **Healthy** | **Healthy** | **Very low overall care use** | **Relatively healthy** | **Mostly healthy** | **Young, healthy** | **Feeling vital** | **Relatively healthy group** | **Relatively healthy group** | **No chronic** | **Robust with no chronic conditions** |
|  | **Adolescents/ paediatrics with No chronic conditions** | **Healthy non-user** | **Mostly healthy adults >75 years old** |  |  |  | **Low primary care use** |  |  | **Middle-age, healthy** |  |  |  |  |  |
|  | **Adults with No chronic conditions** |  |  |  |  |  |  |  |  |  |  |  |  |  |  |
|  | **Elderly with No chronic conditions** |  |  |  |  |  |  |  |  |  |  |  |  |  |  |
|  |  |  |  |  |  |  |  |  |  |  |  |  |  |  |  |
| **Acute illness** |  |  |  |  |  |  |  |  |  |  |  |  |  |  |  |
|  |  | **Major or significant time-limited health needs: Children and Youth < 18 years** |  | **History of significant acute disease** | **Acutely ill** | **Suffering from an acute event** |  |  | **Serious acute illness but curable** |  |  |  |  |  |  |
|  |  | **Major or significant time-limited health needs: Adults** |  |  |  |  |  |  |  |  |  |  |  |  |  |
|  |  |  |  |  |  |  |  |  |  |  |  |  |  |  |  |
| **Maternal and infant health** |  |  |  |  |  |  |  |  |  |  |  |  |  |  |  |
|  |  | **Maternity and healthy newborns** |  |  | **Maternal and infant health** | **Maternity and infancy** |  |  |  |  |  |  |  |  |  |
|  |  |  |  |  |  |  |  |  |  |  |  |  |  |  |  |
| **Minor chronic illness** |  |  |  |  |  |  |  |  |  |  |  |  |  |  |  |
|  | **Infants with 1 chronic condition** | **Low Complex Chronic Conditions** | **Adults (<75) with one or more LTCs** | **Single minor chronic disease** | **Chronic conditions, normal function** | **Possibly affected by chronic disease or at an early stage** | **High primary care use** | **Simple chronic illness** | **Stable chronic** | **Stable, chronic disease** |  |  |  | **Minor chronic** | **One or more chronic conditions** |
|  | **Adolescents/ paediatrics with 1 chronic condition** |  | **Elderly (>75) people with one or more LTCs** | **Minor chronic disease in multiple organ systems** |  | **Affected by only one chronic disease** |  | **Minor complex chronic illness** |  |  |  |  |  |  |  |
|  | **Adults with 1 chronic condition** |  |  |  |  |  |  |  |  |  |  |  |  |  |  |
|  | **Elderly with 1 chronic condition** |  |  |  |  |  |  |  |  |  |  |  |  |  |  |
|  |  |  |  |  |  |  |  |  |  |  |  |  |  |  |  |
| **Moderate chronic illness** |  |  |  |  |  |  |  |  |  |  |  |  |  |  |  |
|  | **Infants with 2+ chronic conditions** | **Medium Complex Chronic Conditions** |  | **Significant chronic disease** | **Stable but serious disability** | **Affected by more than one chronic disease** | **High emergency care use** | **Disabled, aged 65 and below** | **Complex chronic without frequent hospital admissions** | **Complicated chronice disease** | **Physical and mobility complaints** | **Functional Impairment group** | **Physical Impairment** | **Major chronic** |  |
|  | **Adolescents/ paediatrics with 2+ chronic conditions** |  |  | **Significant chronic diseases in multiple organ systems** |  |  | **Specialist care use** |  |  |  |  |  |  |  |  |
|  | **Adults with 2+ chronic conditions** |  |  |  |  |  |  |  |  |  |  |  |  |  |  |
|  | **Elderly with 2+ chronic conditions** |  |  |  |  |  |  |  |  |  |  |  |  |  |  |
|  |  |  |  |  |  |  |  |  |  |  |  |  |  |  |  |
| **Frailty** |  |  |  |  |  |  |  |  |  |  |  |  |  |  |  |
|  |  | **Frail in community with High Complex Chronic Conditions** | **Adults and elderly people with SEMI and FACS eligible physical disability** |  | **Frailty, with or without dementia** | **Elderly** | **High needs but low emergency care use** | **Frail elderly** |  |  | **Feeling extremely frail** | **Frail group** | **Cognitive and physical Impairment** |  |  |
|  |  | **Frail Population, Living in Residential Care** |  |  |  |  |  |  |  |  |  |  |  |  |  |
|  |  | **Frail population, Living in the community** |  |  |  |  |  |  |  |  |  |  |  |  |  |
|  |  |  |  |  |  |  |  |  |  |  |  |  |  |  |  |
| **Major chronic illness** |  |  |  |  |  |  |  |  |  |  |  |  |  |  |  |
|  |  | **High Complex Chronic Conditions without Frail ADL supports** |  | **Dominant chronic disease in 3 or more organ systems** | **Limited reserve and exacerbations** |  | **Very high needs and high emergency care use** | **Major complex chronic illness** | **Complex chronic with frequent hospital admissions** | **Frequent admitters** | **Difficulties experienced in multiple domains** | **High comorbidity group** |  | **Complex chronic** | **Advanced illness and/or end-organ failure** |
|  |  |  |  | **Catastrophic conditions** |  |  |  |  |  |  |  |  |  |  |  |
|  |  |  |  |  |  |  |  |  |  |  |  |  |  |  |  |
| **Cancer** |  |  |  |  |  |  |  |  |  |  |  |  |  |  |  |
|  |  | **Population with Cancer** | **Adults and elderly people with cancer** |  |  |  |  |  |  |  |  |  |  |  |  |
|  |  |  |  |  |  |  |  |  |  |  |  |  |  |  |  |
| **Mental illness** |  |  |  |  |  |  |  |  |  |  |  |  |  |  |  |
|  | **Infants with Mild mental health illness** | **Mental Health and Substance Use Needs** | **Adults and elderly people with severe and enduring mental illness (SEMI)** |  |  |  |  |  |  |  | **Difficulties in psycho-social coping** |  | **Cognitive Impairment** |  |  |
|  | **Infants with Severe mental health illness** |  | **Adults and elderly with advanced organic brain disorders** |  |  |  |  |  |  |  |  |  |  |  |  |
|  | **Adolescents/ paediatrics with Mild mental health illness** |  | **Adults and elderly people with learning disabilities** |  |  |  |  |  |  |  |  |  |  |  |  |
|  | **Adolescents/ paediatrics with Severe mental health illness** |  |  |  |  |  |  |  |  |  |  |  |  |  |  |
|  | **Adults with Mild mental health illness** |  |  |  |  |  |  |  |  |  |  |  |  |  |  |
|  | **Adults with Severe mental health illness** |  |  |  |  |  |  |  |  |  |  |  |  |  |  |
|  | **Elderly with Mild mental health illness** |  |  |  |  |  |  |  |  |  |  |  |  |  |  |
|  | **Elderly with Severe mental health illness** |  |  |  |  |  |  |  |  |  |  |  |  |  |  |
|  |  |  |  |  |  |  |  |  |  |  |  |  |  |  |  |
| **End of life** |  |  |  |  |  |  |  |  |  |  |  |  |  |  |  |
|  |  | **Living in the Community with Palliative Needs** |  | **Dominant and metastatic malignancies** | **Short period of decline before dying** |  | **High needs, emergency and home care use** |  | **End of life** |  |  |  |  |  | **Extreme frailty or near the end of life** |
|  |  |  |  |  |  |  |  |  |  |  |  |  |  |  |  |
| **Excluded subjects** |  |  |  |  |  |  |  |  |  |  |  |  |  |  |  |
|  |  |  | **Adults and elderly people who are socially excluded** |  |  | **Subjects unknown to the system** |  |  |  |  |  |  |  |  |  |
|  |  |  |  |  |  |  |  |  |  |  |  |  |  |  |  |
| **Number of segment themes** | 4 | 10 | 6 | 6 | 8 | 7 | 7 | 5 | 6 | 4 | 5 | 4 | 4 | 4 | 4 |

Comment: Segments were grouped together according to conceptually similar underlying healthcare needs. The Johns Hopkins ACG System population segmentation scheme is described separately in its technical reference guide (2). There are 8 segment themes included by the ACG namely: healthy, acute illness, maternal and infant health, minor chronic disease, moderate chronic disease, major chronic disease, mental illness, and excluded subjects. Segmentation tools with a larger number of segment themes may potentially have a higher ‘actionability’ potential.

## **Appendix D**

| Reference | Author(s), year | Segmentation Tool | District / County; Country | Target population | Data Source | Statistical Model validation | Sample Size | QUIPS overall risk of bias | Commentary |
| --- | --- | --- | --- | --- | --- | --- | --- | --- | --- |
| (3) | Starfield and Mumford, 1991 | ACG | USA | Health maintenance organization and Medicaid enrollee | Secondary data: Four different HMOs and a large Medicaid population: Columbia Medical Plan, Maxicare, MedCenters Health Plan, Harvard Community Health Plan, and claims for over 30,000 continuously eligible Medicaid recipients in Baltimore, Maryland | No | 160,000 | Low | Regression on ambulatory visits and charges for a model consisting of 51 ACG variables found the adjusted R2 to range from 0.34 to 0.50 for same year visits and 0.18-0.20 for subsequent year visits. This is superior to a model which consists only of age and gender for which adjusted R2 ranged from 0.03 to 0.06 for both years. |
| (4) | Juncosa et al, 1999 | ACG | Barcelona, Spain | Patients under doctors who voluntarily participated | Primary data collection | No | 2,467 | Low | Using ordinary least square regressions and log-transformed dependent variables, ACGs explained 64% variance (R2) for the number of episodes per patient, 44% for visits per patient, 41% for primary care costs and 31% for total costs. (Table 2, compared with ADG and demographic) |
| (5) | Reid et al, 2001 | ACG | Manitoba & British Columbia, Canada | All residents who were continuously enrolled in the provincial health plans from April 1, 1995, to March 31, 1997. | Secondary data: databases of universal health care insurance programs | Yes | 454,7397 | Low | In linear regressions, ACGs explained 31-47% and 8-23% of the variation (R2) in concurrent year and prospective year truncated physician costs, respectively; 15-37% and 3-11% of same-year and next-year total costs respectively; depending on target age group. (Table 1, compared to ADG and demographic) |
| (6) | Sicras-Mainar, 2008 | ACG | Catalonia, Spain | Patients under five primary care teams (PCT) and two hospitals during the period of one year (2005). | Secondary data: clinical records | No | 81,873 | Low | ACG classification explained (R2) 73.1% variance (75.5% log-transformed) of episodes, 43.2% (54.0% log-transformed) of visits, 19.6% (54.8% log-transformed) of primary care costs, and 22.7% (48.3% log-transformed) of total costs, p=0,000. (from text, no table) |
| (7) | Zielinski et al, 2009 | ACG | Blekinge county, Sweden | non-private patients | Secondary data: Electronic Primary health care records of inhabitants in Blekinge County | No | 120,000 | Low | Linear regression models on cost which originally included only age, gender and listing with specifc primary healthcare provider demonstrated an adjusted R2 increase from approximately 0.14 to 0.60 when information on level of co-morbidity as measured by ACG was added. |
| (8) | Haas et al, 2013 | ACG | Rochester, Minnesota, US | All primary care patients 18 years or older empaneled to the Employee and Community Health (ECH) practice (family medicine, primary care internal medicine, and community pediatric and adolescent medicine) | Secondary data: electronic medical record and administrative databases within Mayo Clinic’s health records system | No | 83,187 | Low | The authors used logistic regressions to compare ACGs with 5 other risk-adjustment measures in predicting hospitalizations, ED visits and top 10% highest cost users. ACG outperformed 5 other risk-adjustment measures for all dependent variables (c-statistic 0.73 for hospitalization, 0.67 for ED visits, 0.81 for readmission and 0.76 for top 10% highest cost users.) (Table 2) |
| (9) | Lemke, Weiner, and Clark, 2012 | ACG | USA | Individuals <65 years old in employer-sponsored or private health plans, individuals ≥ 65 years old Medicare beneficiaries in managed care plans. | Secondary data: IMS Health Plan Claims Database which includes private health plans contracted with employers, private individuals, Medicaid and Medicare. | Yes | 9,339,227 | Low | Multivariable logistic regression on hospitalization outcomes demonstrated superior performance of an ACG hospitalization model (AUC = 0.8) compared to a non-ACG hospitalization model (AUC=0.75) and Charlson comorbidity hospitalization model (AUC = 0.78) |
| (10) | Bolanos-Carmona et al, 2002 | ACG 4 | Spain | Patients under 38 primary care physicians working in 21 health centres | Primary data collection | No | 52,152 | Low | In multilevel regression, ACGs explained 49% variability (adjusted R2) of number of physician visits, 14% variability of referrals to specialists, 16% variability of number of diagnostic tests. (ACG only) |
| (11) | Wahls, Barnett, and Rosenthal, 2004 | ACG 4.5 | Iowa & Nebraska, US | Patients who had one or more primary care visits at 4 hospital and 19 community-based VA facilities and received one or more prescriptions in fiscal year 1999 (October 1999 through September 2000). | Secondary data: VA Pharmacy Benefits Management database (PBM), the Patient Treatment File (PTF), and the Outpatient Care File (OPC) | No | 31,212 | Low | ACGs (adjusted for age and gender) were predictive of total outpatient visits, hospitalization and total hospital days. In ordinary least square regression, ACGs explained 30.2% variance (adjusted R2) of total outpatient visits in the same year and 16.3% variance in the subsequent year. C-statistics were 0.86 and 0.84 for same-year hopitalizations and same year total hospital days; 0.72 and 0.71 for subsequent year hopitalization and total hospital days. (Table 2 & 3, compared with CDI and abridged ACG) |
| (12) | Pietz et al, 2004 | ACG 5 | US | Primary care patients in a network of 8 VA hospitals and their associated clinics in the Pacific Northwest | Secondary data: electronic medical database | Yes | 78,344 | Low | Using weighted least square regressions, ACGs explained 25.3% variance (adjusted R2) of current year costs in training dataset and 27.7% in testing dataset; 8.7% prospective year costs in training dataset and 7% in testing dataset. (compared to ADGs and demographics) |
| (13) | Orueta et al, 2006 | ACG 5 | Spain | All patients under 46 general practitioners and 10 pediatricians from primary health care centers of the Osakidetza / Basque Health Service. | Secondary data: medical record form for primary care of the Osakidetza / Basque Health Service | No | 84,136 | Low | In multiple linear regressions, the ACGs system explained (adjusted R2) 53% of the variance in physician visits, 31-32% of prescriptions, 24% of referrals, 25-26% requests of laboratory tests, and 14% of radiographs. (Table 5, compared to ADGs and demographics) |
| (14) | Hanley, Morgan and Reid, 2010 | ACG 7 | British Columbia, Canada | Residents who were registered for the universal health care plan for 275 days in the calendar years 2004 and 2005. | Secondary data: For every resident in our cohort, we obtained demographic information including age, sex, and the geographic locale of residence (Local Health Area) from the Medical Services Plan registration files. We obtained diagnostic codes, (ICD-9-CM and ICD-10 codes) for the study population from administrative records of hospital separations and physician paid claims records during the calendar year 2004. | Yes | 3,908,533 | Low | The C-statistics from logistic models explaining pharmaceutical use for concurrent (2004 diagnoses predicting 2004 use)/ prospective (2004 diagnoses predicting 2005 use) models were 0.87/0.82 for the ACG model compared with 0.78/0.76 for the Charlson index model, and 0.75/0.75 for the age and sex model. |
| (15) | Aguado et al, 2008 | ACG 7.1 | Spain | Adult & pediatric patients | Secondary data: electronic records of five primary care centers in Spain | No | 65,630 | Moderate | Authors modeled pharmaceutical expenditures using two-part model (logistic regression and linear mixed model), adjusting for age. In the adult population, ACG explained 28.8% variance of incurrence of pharmaceutical expenditure and 35.4% of expediture level whereas in the pediatric population, ACG explained 20.6% variance of incurrence of expenditure and 22.4% variance of level of expenditure. (Table 4) |
| (16) | Chang and Weiner, 2010 | ACG 7.1 | Taiwan | Taiwanese National Health Insurance enrollee | Secondary data: Longitudinal dataset prepared by Taiwan’s BNHI; A random sample (1% of Taiwan's population) of Taiwanese National Health Insurance (NHI) enrollees was selected. Those continuously enrolled in 2002 were included for concurrent analyses (n = 173,234), while those in both 2002 and 2003 were included for prospective analyses (n = 164,562). | Yes | 337,796 | Low | The adjusted R2 of total healthcare expenditure in concurrent/prospective multivariate linear regression analyses were 0.04/0.04 in the demographic model compared with 0.15/0.09 in the ACG only model. |
| (17) | Sicras-Mainar, 2012 | ACG 8.2 | Catalonia, Spain | Patients registered in study centres in 2008 | Secondary data: All patients registered in the study centres who required care between 1 January and 31 December 2008 | No | 227,235 | Moderate | The adjusted R2 of the ACG model for cost per patient treated during the study period was 0.37 (0.57 without outliers). |
| (18) | Brilleman et al, 2014 | ACG 8.2 | England | Patients ≥20 years old in 174 practices | Secondary data: General Practice Research Database which is broadly representative of the general UK population | No | 85,946 | Low | Both linear and exponential models were more predictive for total healthcare cost when AGC variables were included on a model which included age, gender, deprivation and practice. The log model R2_D was 0.41 vs 0.23 while R2_COR was 0.27 vs 0.14. Meanwhile for the linear model, R2 was 0.27 vs 0.14. |
| (1) | Orueta et al, 2013 | ACG 9 | Basque Country, Spain | Citizens over 14 years of age | Secondary data: Osakidetza. The study period corresponds to two consecutive 12-month intervals. Data from the first year were used to develop the explanatory variables and those from the second year for the response variables. | Yes | 1,964,337 | Low | Logistic regression models to identify patients located above the 95th percentile of health spending found the ACG (diagnosis), age, gender model (AUC = 0.85) superior to a model with only age and gender (AUC = 0.77) |
| (19) | Hughes et al, 2004 | CRG | USA | Medicare, Medicaid and Private Insurance enrollee | Secondary data: A 5% sample of Medicare enrollees for years 1991-1994,a privately insured population enrolled during the same time period, and a Medicaid population with 2 years of data. | Yes | 1,286,574 | Low | Regression models to predict medical expenditure in the prospective Medicare validation dataset demonstrated a R2 value of 0.12 without adjusting predicted payments for persons who died in the prediction year. A concurrent analysis, using diagnostic information from the same year as expenditure, yielded an R2 of 0.43. |
| (20) | Garcia-Goni and Ibern, 2006 | CRG | Catalonia, Spain | For inpatient services, population covered by the integrated delivery system can use the only hospital in the county – Palamós Hospital, while for outpatient services there are five different primary care areas. | Secondary data: Electronic database | No | 87,691 | Low | CRGs alone explained 23% variance (adjusted R2) current-year total costs and 19% variance of next-year total costs. When demographic information was added, total variance explained ranged from 18 - 25% for current-year total and 16 - 22% for next-year total costs in different model types. |
| (1) | Orueta et al, 2013 | CRG | Basque Country, Spain | Citizens > 14 years old | Secondary data: Osakidetza. The study period corresponds to two consecutive 12-month intervals. Data from the first year were used to develop the explanatory variables and those from the second year for the response variables. | Yes | 1,964,337 | Low | Logistic regression models to identify patients located above the 95th percentile of health spending found the CRG (diagnosis), age, gender model (AUC = 0.8) superior to a model with only age and gender (AUC = 0.77) |
| (21) | Vivas-Consuelo et al, 2014 | CRG | Valencia Community, Spain | Population in one health district of an Eastern region of Spain | Secondary data: Population Information System’s (PIS) database. Data for the study was obtained from the electronic health record for primary health care (SIA) and the Minimum Data Set (MDS) of hospitals. Data for primary health care pharmaceutical expenditure was obtained from the prescription module of the Pharmaceutical Provision Manager, GAIA. | No | 4,700,000 | Moderate | The CRG core health status based regression model (R2 = 0.55) is superior to a model based only on age and gender (R2 = 0.28) for explaining pharmaceutical expenditure. Significant differences were observed between the predictive budget using the model developed and real spending in some health districts. |
| (22) | Vivas-Consuelo et al, 2014 | CRG | Valencia Community, Spain | Population in one health district of an Eastern region of Spain | Secondary data: Data for each patient included in the study was obtained from the Ambulatory Information System which was integrated in the EHR of the Valencia Health Agency of the Generalitat Valenciana (Autonomous Health Department). The Population Information System (PIS) from the Valencia Health Agency provided demographic information. | No | 261,054 | Moderate | The R2 of the WHO-ATC regression model was 0.53 while that of the CRG regression model was 0.48 when utilized to predict pharmaceutical spending thus suggesting that both have similar predictive validity. |
| (23) | Fuller, Hughes and Goldfield, 2016 | CRG | US | Enrollee 2006 to 2007 for training; enrollee 2010 to 2011 for validation | Secondary data: Medicare claims processing data | No | 28,883,443 | Moderate | Using multivariate regression, CRGs alone explained 25% variance (R2) natural logged claims payment and 27% when including adjustments for functional groups. Limiting the analysis only to those with functional groups yielded R2 of 14% (CRG alone) and 15% (CRG with adjustments). |
| (24) | Eissens van der Laan et al, 2014 | Demand-driven care model | Netherlands | Adults 65 - 101 years old | Primary data collection | No | 2,019 | High | Authors attempted to validate their data-driven segments by illustrating the increase in the number of care providers (general practitioners, medical specialists, physiotherapists, dieticians, psychosocial care provider, nursing home doctors, help in retirement / nursing homes) as the intensity of the experienced difficulties increases. |
| (25) | Liu, 2012 | LCA of NHIS Taiwan | Taiwan | 65 years old and older | Secondary data: Taiwan National Health Interview Survey (NHIS) 2005; National Health Insurance Research Database (NHIRD) 2004 - 2007 | Yes | 3,940 | Moderate | Authors used logistic regression to validate their data-driven segments by examining their associations with being heavy user of ambulatory care and inpatient care. For instance, “High Comorbidity” segment had a greater effect (OR = 3.342, p < 0.001) on the likelihood of heavy utilization of ambulatory care services (beyond 15 visits annually) than did the other health segments. In addition, authors also ran ordinary least square regressions on log-transformed costs and found that being in "high comorbidity", "frail", "functional impairment" segments were significantly associated with higher costs compared to "relatively healthy" segment. No model fitting test was reported. |
| (26) | Lafortune et al, 2009 | LCA of SIPA | Canada | Elderly 64 years old and older | Secondary data: Canada System of Integrated Care for the frail elderly (SIPA) 1999-2001 | No | 1,164 | Moderate | Authors used two-part model and utilization costs of health and social services (e.g. nursing homes, acute hospital, medical visits, etc) to validate its data-driven segments. R2 ranged from 3% to 14%. |
| (27) | Zhou, Wong, and Li, 2014 | Senior Segmentation Algorithm | USA | Kaiser Permanente managed care enrollee ≥65 years old | Secondary data: Kaiser Permanente Northwest and Hawaii Databases; | No | 91,113 | High | Concordance of the algorithm with physician assessed segmentation of 1615 Medicare recipients was 85%. After 1 year, approximately 85% of 86,140 surviving seniors remained in the same care group; 3.9% moved to a lower need group; and 11% moved to a higher need group. Six-month and 12-month mortality rates varied substantially across care groups. The algorithm performed similarly to the likelihood of hospitalization score in predicting hospitalization and readmissions. |
| (28) | Joynt et al, 2017 | Joynt et al’s Medicare claims based segmentation | USA | Medicare beneficiaries | Secondary data: 2011-2012 Medicare Beneficiary Denominator and Enrollment Database, Carrier File, Inpatient File, Outpatient File, Skilled Nursing, Home Health and Hospice, and Durable Medical Equipment Files, Part D File, and Impact File | No | 6,112,450 | High | Authors assigned high-cost patients and non-high-cost patients to the population segments before computing the segment specific risk of being designated a high-cost beneficiary. Within each segment, the risk of being designated a high-cost beneficiary varied markedly, with frail patient being the most likely (46.2%) followed by the under-65 disabled population (14.3%). |
| (29) | Low et al, 2018 | Low et al’s utilization based segmentation | Singapore | Singapore residents who resided in and had a healthcare encounter with the Singhealth Regional Health System in 2012 | Secondary data: Singapore Health Services Electronic Health Intelligence System (eHints), an electronic medical record database that included Healthcare utilization (inpatient admissions, specialist outpatient clinic visits, emergency department visits, and primary care clinic visits), mortality and diseases and demographic details | No | 146,999 | Moderate | Authors applied hierarchical clustering analysis (Ward’s linkage) and K-means cluster analysis using age and healthcare utilization data in 2012 to segment the selected population. The segments were then evaluated in terms of morbidity/mortality and longitudinal healthcare utilization from 2013-2016. 5 Segments were created. The “Frequent admitters” segment notably had the smallest number of patients (1.79% of the population) but utilized 69% of inpatient admissions. |
| (30) | Low et al, 2017 | Singapore MOH Segmentation Framework | Singapore | Singapore residents who resided in and had a healthcare encounter with the Singhealth Regional Health System in 2012 | Secondary data: Singapore Health Services Electronic Health Intelligence System (eHints) | No | 825,874 | High | Authors examined and compared patient demographics, prevalence of chronic diseases and hospital health services utilization using Chi-square tests for categorical variables and one-way ANOVA test for continuous variables for patients in the different population segments. Patients in the category of ‘complex chronic disease with frequent hospital admissions’ accounted for the highest hospital admissions and emergency attendances per patient and had a high mortality rate. |

**Appendix E**

**Adaptation of QUIPS tool for evaluation of segmentation tool validation studies**

|  | **Variable** | **Adaptation for evaluation of segmentation tool validation studies** |
| --- | --- | --- |
| 1 | **Quips1a_adequate participation in the study by eligible persons** | Adequate participation is when the entire population in a geographical location is included without age/gender exclusion |
|  | **Quips1b_description of the source population of population of interest** | Describes how the data was obtained. E.g. Administrative records, etc |
|  | **Quips1c_description of the baseline study sample** | Descriptive table exists which describes baseline characteristics |
|  | **Quips1d_adequate description of the sampling frame and recruitment** |  |
|  | **Quips1e_adequate description of the period and place of recruitment** | Describes where the population resides geographically and temporally |
|  | **Quips1f_adequate description of inclusion and exclusion criteria** |  |
| 2 | **Quips2a_adequate response rate for study participants** | Cross-sectional studies are not applicable. Otherwise, response rate should be >80% |
|  | **Quips2b_description of attempts to collect information on participants who dropped out** | Cross-sectional studies are not applicable |
|  | **Quips2c_reasons for loss to follow-up are provided** | Cross-sectional studies are not applicable |
|  | **Quips2d_adequate description of participants lost to follow-up** | Cross-sectional studies are not applicable |
|  | **Quips2e_there are no important differences between participants who completed the study and those who did not** | Cross-sectional studies are not applicable |
| 3 | **Quips3a_a clear definition or description of the prognostic factor is provided** | Segmentation scheme definition included in paper or relevant citations given to allow reproducibility |
|  | **Quips3b_method of prognostic factor measurement is adequately valid and reliable** | All schemes are shortlisted because there is a validation component |
|  | **Quips3c_continuous variables are reported or appropriate cut points are used** |  |
|  | **Quips3d_the method and setting of measurement of prognostic factor is the same for all study participants** | Not relevant unless different schemes are used, or different data used to feed into same segmentation algorithm. |
|  | **Quips3e_adequate proportion of the study sample has complete data for the prognostic factor** | Adequate if complete information available for input into segmentation mechanism > 80% of sample. |
|  | **Quips3f_appropriate methods of imputation are used for missing prognostic factor data** |  |
| 4 | **Quips4a_a clear definition of the outcome is provided** |  |
|  | **Quips4b_method of outcome measurement used is adequately valid and reliable** |  |
|  | **Quips4c_ the method and setting of outcome measurement is the same for all study participants** | If > 80% reported having complete outcome information, consider adequate. |
|  | **Quips4d_same method of assessment at follow-up** |  |
| 5 | **Quips5a_all important confounders are measured** | Must include age and gender to be yes |
|  | **Quips5b_clear definition of the important confounders measured are provided** | Age and gender are self-explanatory |
|  | **Quips5c_measurement of all important confounders is adequately valid and reliable** | Age and gender are self-explanatory |
|  | **Quips5d_ the method and setting of confounding measurement are the same for all study participants** | Age and gender are self-explanatory |
|  | **Quips5e_appropriate methods are used if imputation is used for missing confounder data** |  |
|  | **Quips5f_important potential confounders are accounted for in the study design and analysis** | Age and gender |
| 6 | **Quips6a_sufficient presentation of data to assess adequacy of analytic strategy** | Deemed sufficient if analysis results presented at least in table form |
|  | **Quips6b_strategy for model building is appropriate and is based on a conceptual framework or model** | All segmentation tools are expert, or data driven thus follow conceptual or model framework. |
|  | **Quips6c_the selected statistical model adequate for design of study** | Adequate if statistical analysis demonstrates prognostic ability of chosen outcome |
|  | **Quips6d_there is no selective reporting of results** | Prognostic ability of all segments should ideally be described, therefore reporting of variance explained measured such as R2 and BIC are acceptable. If certain segments omitted from reported analysis, e.g. ANOVA analysis conducted omitted some segments' prognostic ability, consider as selective reporting. |

**Appendix F: Detailed QUIPS rating**

| S/N | First Author | QUIPS1a_AdeParticipation | QUIPS1b_DescSource | QUIPS1c_DescBaseline | QUIPS1d_DescFrameRecruit | QUIPS1e_DescPeriodPlace | QUIPS1f_DesInExCriteria | QUIPS2a_ResponseRate | QUIPS2b_AttempDropOut | QUIPS2c_ReasonsLoss | QUIPS2d_DescDropOut | QUIPS2e_NoDiffDropOut | QUIPS3a_ClearPF | QUIPS3b_MeasurePF | QUIPS3c_ReportContCutOff | QUIPS3d_SameMethodParticipants | QUIPS3e_AdeComplete | QUIPS3f_ApproImputation | QUIPS4a_ClearOutcome | QUIPS4b_MeasureOutcome | QUIPS4c_SameMethodParticipants | QUIPS4d_SameMethodFollowUp |
| --- | --- | --- | --- | --- | --- | --- | --- | --- | --- | --- | --- | --- | --- | --- | --- | --- | --- | --- | --- | --- | --- | --- |
| 1 | ORUETA | No | Yes | Yes | Yes | Yes | Yes | Not Applicable | Not Applicable | Not Applicable | Not Applicable | Not Applicable | Yes | Yes | Yes | Yes | No | Yes | Yes | Yes | Yes | Yes |
| 3 | STARFIELD | Yes | Yes | No | Yes | Yes | Yes | Not Applicable | Not Applicable | Not Applicable | Not Applicable | Not Applicable | Yes | Yes | Yes | Yes | Yes | Yes | Yes | Yes | Yes | Yes |
| 4 | JUNCOSA | Yes | Yes | No | Yes | Yes | Yes | Not Applicable | Not Applicable | Not Applicable | Not Applicable | Not Applicable | Yes | Yes | Yes | Yes | Yes | Yes | Yes | Yes | Yes | Yes |
| 5 | REID | Yes | Yes | Yes | Yes | Yes | Yes | Not Applicable | Not Applicable | Not Applicable | Not Applicable | Not Applicable | Yes | Yes | Yes | Yes | Yes | Yes | Yes | Yes | Yes | Yes |
| 6 | SICRAS-MAINAR | Yes | Yes | Yes | Yes | Yes | Yes | Not Applicable | Not Applicable | Not Applicable | Not Applicable | Not Applicable | Yes | Yes | Yes | Yes | Yes | Yes | Yes | Yes | Yes | Yes |
| 7 | ZIELINSKI | Yes | Yes | Yes | Yes | Yes | Yes | Not Applicable | Not Applicable | Not Applicable | Not Applicable | Not Applicable | Yes | Yes | Yes | Yes | Yes | Yes | Yes | Yes | Yes | Yes |
| 8 | HAAS | No | Yes | Yes | Yes | Yes | Yes | Not Applicable | Not Applicable | Not Applicable | Not Applicable | Not Applicable | Yes | Yes | Yes | Yes | Yes | Yes | Yes | Yes | Yes | Yes |
| 9 | LEMKE | No | Yes | Yes | Yes | Yes | Yes | Not Applicable | Not Applicable | Not Applicable | Not Applicable | Not Applicable | Yes | Yes | Yes | Yes | Yes | Yes | Yes | Yes | Yes | Yes |
| 10 | BOLANOS-CARMONA | Yes | Yes | Yes | Yes | Yes | Yes | Not Applicable | Not Applicable | Not Applicable | Not Applicable | Not Applicable | Yes | Yes | Yes | Yes | Yes | Yes | Yes | Yes | Yes | Yes |
| 11 | WAHLS | No | Yes | Yes | Yes | Yes | Yes | Not Applicable | Not Applicable | Not Applicable | Not Applicable | Not Applicable | Yes | Yes | Yes | Yes | No | Yes | Yes | Yes | Yes | Yes |
| 12 | PIETZ | No | Yes | Yes | Yes | Yes | Yes | Not Applicable | Not Applicable | Not Applicable | Not Applicable | Not Applicable | Yes | Yes | Yes | Yes | Yes | Yes | Yes | Yes | Yes | Yes |
| 13 | ORUETA | Yes | Yes | Yes | Yes | Yes | Yes | Not Applicable | Not Applicable | Not Applicable | Not Applicable | Not Applicable | Yes | Yes | Yes | Yes | Yes | Yes | Yes | Yes | Yes | Yes |
| 14 | HANLEY | Yes | Yes | Yes | Yes | Yes | Yes | Not Applicable | Not Applicable | Not Applicable | Not Applicable | Not Applicable | Yes | Yes | Yes | Yes | Yes | Yes | Yes | Yes | Yes | Yes |
| 15 | AGUADO | Yes | Yes | Yes | Yes | Yes | Yes | Not Applicable | Not Applicable | Not Applicable | Not Applicable | Not Applicable | Yes | Yes | Yes | Yes | No | Yes | Yes | Yes | Yes | Yes |
| 16 | CHANG | Yes | Yes | Yes | Yes | Yes | Yes | Not Applicable | Not Applicable | Not Applicable | Not Applicable | Not Applicable | Yes | Yes | Yes | Yes | Yes | Yes | Yes | Yes | Yes | Yes |
| 17 | SICRAS-MAINAR | Yes | Yes | Yes | Yes | Yes | Yes | Not Applicable | Not Applicable | Not Applicable | Not Applicable | Not Applicable | Yes | Yes | Yes | Yes | Yes | Yes | Yes | Yes | Yes | Yes |
| 18 | BRILLEMAN | No | Yes | Yes | Yes | Yes | Yes | Not Applicable | Not Applicable | Not Applicable | Not Applicable | Not Applicable | Yes | Yes | Yes | Yes | Yes | Yes | Yes | Yes | Yes | Yes |
| 19 | HUGHES | No | Yes | Yes | Yes | Yes | Yes | Not Applicable | Not Applicable | Not Applicable | Not Applicable | Not Applicable | Yes | Yes | Yes | Yes | No | Yes | Yes | Yes | Yes | Yes |
| 20 | GARCIA-GONI | No | Yes | Yes | Yes | Yes | Yes | Not Applicable | Not Applicable | Not Applicable | Not Applicable | Not Applicable | Yes | Yes | Yes | Yes | No | Yes | Yes | Yes | Yes | Yes |
| 21 | VIVAS-CONSUELO | Yes | Yes | Yes | Yes | Yes | Yes | Not Applicable | Not Applicable | Not Applicable | Not Applicable | Not Applicable | Yes | Yes | Yes | Yes | No | Yes | Yes | Yes | Yes | Yes |
| 22 | VIVAS-CONSUELO | Yes | Yes | Yes | Yes | Yes | Yes | Not Applicable | Not Applicable | Not Applicable | Not Applicable | Not Applicable | Yes | Yes | Yes | Yes | No | Yes | Yes | Yes | Yes | Yes |
| 23 | FULLER | No | Yes | Yes | Yes | Yes | Yes | Not Applicable | Not Applicable | Not Applicable | Not Applicable | Not Applicable | Yes | Yes | Yes | Yes | No | Yes | Yes | Yes | Yes | Yes |
| 24 | VAN DER LAAN | No | Yes | Yes | Yes | Yes | Yes | Not Applicable | Not Applicable | Not Applicable | Not Applicable | Not Applicable | Yes | Yes | Yes | Yes | No | Yes | Yes | Yes | Yes | Yes |
| 25 | LIU | No | Yes | Yes | Yes | Yes | Yes | No | No | No | Yes | No | Yes | Yes | Yes | Yes | Yes | Yes | Yes | Yes | Yes | Yes |
| 26 | LAFORTUNE | No | Yes | Yes | Yes | Yes | Yes | Yes | No | No | No | Not Applicable | Yes | Yes | Yes | Yes | Yes | Yes | Yes | Yes | Yes | Yes |
| 27 | ZHOU | No | Yes | Yes | Yes | Yes | Yes | No | No | No | No | No | Yes | Yes | Yes | Yes | No | Yes | Yes | Yes | Yes | Yes |
| 28 | JOYNT | No | Yes | Yes | Yes | Yes | Yes | Not Applicable | Not Applicable | Not Applicable | Not Applicable | Not Applicable | Yes | Yes | Yes | Yes | Yes | Yes | Yes | Yes | Yes | Not Applicable |
| 29 | LOW | No | Yes | Yes | Yes | Yes | Yes | Yes | Not Applicable | Not Applicable | Not Applicable | Not Applicable | Yes | Yes | Yes | Yes | Yes | Yes | Yes | Yes | Yes | Yes |
| 30 | LOW | No | Yes | Yes | Yes | Yes | Yes | Not Applicable | Not Applicable | Not Applicable | Not Applicable | Not Applicable | Yes | Yes | Yes | Yes | No | Yes | Yes | Yes | Yes | Yes |

| S/N | First Author | QUIPS5a_MeasureConfounders | QUIPS5b_DefineConfounders | QUIPS5c_MethodsConfounders | QUIPS5d_SameMethod | QUIPS5e_ApproImputation | QUIPS5f_AccountConfounders | QUIPS6a_SufficientPresentation | QUIPS6b_Strategy | QUIPS6c_ModelAdequate | QUIPS6d_SelectiveReporting | Q1Participation | Q2Attrition | Q3ProgMeasure | Q4OutcomeMeasure | Q5Confounding | Q6StatsReport | Overall risk of bias |
| --- | --- | --- | --- | --- | --- | --- | --- | --- | --- | --- | --- | --- | --- | --- | --- | --- | --- | --- |
| 1 | ORUETA | Yes | Yes | Yes | Yes | No | Yes | Yes | Yes | Yes | Yes | Unlikely | Unlikely | Unlikely | Unlikely | Unlikely | Unlikely | **LOW** |
| 3 | STARFIELD | Yes | Yes | Yes | Yes | Yes | No | Yes | Yes | Yes | Yes | Unlikely | Unlikely | Unlikely | Unlikely | Unlikely | Unlikely | **LOW** |
| 4 | JUNCOSA | Yes | Yes | Yes | Yes | No | Yes | Yes | Yes | Yes | Yes | Unlikely | Unlikely | Unlikely | Unlikely | Unlikely | Unlikely | **LOW** |
| 5 | REID | Yes | Yes | Yes | Yes | No | Yes | Yes | Yes | Yes | Yes | Unlikely | Unlikely | Unlikely | Unlikely | Unlikely | Unlikely | **LOW** |
| 6 | SICRAS-MAINAR | Yes | Yes | Yes | Yes | No | Yes | Yes | Yes | Yes | Yes | Unlikely | Unlikely | Unlikely | Unlikely | Unlikely | Unlikely | **LOW** |
| 7 | ZIELINSKI | Yes | Yes | Yes | Yes | No | Yes | Yes | Yes | Yes | Yes | Unlikely | Unlikely | Unlikely | Unlikely | Unlikely | Unlikely | **LOW** |
| 8 | HAAS | Yes | Yes | Yes | Yes | No | Yes | Yes | Yes | Yes | Yes | Unlikely | Unlikely | Unlikely | Unlikely | Unlikely | Unlikely | **LOW** |
| 9 | LEMKE | Yes | Yes | Yes | Yes | No | Yes | Yes | Yes | Yes | Yes | Unlikely | Unlikely | Unlikely | Unlikely | Unlikely | Unlikely | **LOW** |
| 10 | BOLANOS-CARMONA | Yes | Yes | Yes | Yes | No | Yes | Yes | Yes | Yes | Yes | Unlikely | Unlikely | Unlikely | Unlikely | Unlikely | Unlikely | **LOW** |
| 11 | WAHLS | Yes | Yes | Yes | Yes | No | Yes | Yes | Yes | Yes | Yes | Unlikely | Unlikely | Unlikely | Unlikely | Unlikely | Unlikely | **LOW** |
| 12 | PIETZ | Yes | Yes | Yes | Yes | No | Yes | Yes | Yes | Yes | Yes | Unlikely | Unlikely | Unlikely | Unlikely | Unlikely | Unlikely | **LOW** |
| 13 | ORUETA | Yes | Yes | Yes | Yes | No | Yes | Yes | Yes | Yes | Yes | Unlikely | Unlikely | Unlikely | Unlikely | Unlikely | Unlikely | **LOW** |
| 14 | HANLEY | Yes | Yes | Yes | Yes | No | Yes | Yes | Yes | Yes | Yes | Unlikely | Unlikely | Unlikely | Unlikely | Unlikely | Unlikely | **LOW** |
| 15 | AGUADO | Yes | Yes | Yes | Yes | No | Yes | Yes | Yes | Yes | Yes | Maybe | Unlikely | Unlikely | Unlikely | Unlikely | Unlikely | **MODERATE** |
| 16 | CHANG | Yes | Yes | Yes | Yes | No | Yes | Yes | Yes | Yes | Yes | Unlikely | Unlikely | Unlikely | Unlikely | Unlikely | Unlikely | **LOW** |
| 17 | SICRAS-MAINAR | Yes | Yes | Yes | Yes | No | Yes | No | Yes | Yes | No | Unlikely | Unlikely | Unlikely | Unlikely | Unlikely | Maybe | **MODERATE** |
| 18 | BRILLEMAN | Yes | Yes | Yes | Yes | No | Yes | Yes | Yes | Yes | Yes | Unlikely | Unlikely | Unlikely | Unlikely | Unlikely | Unlikely | **LOW** |
| 19 | HUGHES | Yes | Yes | Yes | Yes | No | Yes | Yes | Yes | Yes | Yes | Unlikely | Unlikely | Unlikely | Unlikely | Unlikely | Unlikely | **LOW** |
| 20 | GARCIA-GONI | Yes | Yes | Yes | Yes | No | Yes | Yes | Yes | Yes | Yes | Unlikely | Unlikely | Unlikely | Unlikely | Unlikely | Unlikely | **LOW** |
| 21 | VIVAS-CONSUELO | Yes | Yes | Yes | Yes | No | Yes | Yes | Yes | Yes | Yes | Unlikely | Unsure | Unlikely | Unlikely | Unlikely | Unlikely | **MODERATE** |
| 22 | VIVAS-CONSUELO | No | Yes | Yes | Yes | No | No | Yes | Yes | Yes | Yes | Unlikely | Unlikely | Unlikely | Unlikely | Maybe | Unlikely | **MODERATE** |
| 23 | FULLER | No | No | Yes | Yes | No | No | Yes | Yes | Yes | Yes | Unlikely | Unlikely | Unlikely | Unlikely | Maybe | Unlikely | **MODERATE** |
| 24 | VAN DER LAAN | No | Not Applicable | Not Applicable | Not Applicable | Not Applicable | Not Applicable | Yes | No | No | Yes | Unlikely | Unlikely | Unlikely | Unlikely | Very likely | Maybe | **HIGH** |
| 25 | LIU | Yes | Yes | Yes | Yes | No | Yes | Yes | Yes | Yes | Yes | Unlikely | Maybe | Unlikely | Unlikely | Unlikely | Unlikely | **MODERATE** |
| 26 | LAFORTUNE | Yes | Yes | Yes | Yes | Not Applicable | Yes | Yes | Yes | Yes | Yes | Maybe | Maybe | Unlikely | Unlikely | Unlikely | Unlikely | **MODERATE** |
| 27 | ZHOU | No | Not Applicable | Not Applicable | Not Applicable | Not Applicable | Yes | Yes | Not Applicable | Not Applicable | Not Applicable | Very likely | Very likely | Unlikely | Unlikely | Very likely | Very likely | **HIGH** |
| 28 | JOYNT | No | No | No | No | No | No | Yes | Yes | No | Yes | Unlikely | Unlikely | Unlikely | Unlikely | Very likely | Maybe | **HIGH** |
| 29 | LOW | Yes | Yes | Yes | Yes | No | Yes | Yes | No | Yes | Yes | Unlikely | Unlikely | Unlikely | Unlikely | Unlikely | Maybe | **MODERATE** |
| 30 | LOW | No | No | No | No | No | No | Yes | Yes | Yes | No | Unlikely | Unlikely | Unlikely | Unlikely | Very likely | Maybe | **HIGH** |

**References**

1. Orueta JF, Nuno-Solinis R, Mateos M, Vergara I, Grandes G, Esnaola S. Predictive risk modelling in the Spanish population: a cross-sectional study. BMC Health Serv Res. 2013;13:269.

2. Department_of_Health_Policy_and_Management. The Johns Hopkins ACG System Version 11 Technical Reference Guide2015 8th July 2018. Available from: <https://www.healthpartners.com/ucm/groups/public/@hp/@public/documents/documents/cntrb_035024.pdf>.

3. Starfield B, Mumford L. Ambulatory Care Groups: A Categorization of Diagnoses for Research and Management. Health Services Research. 1991;26(1):53-74.

4. Juncosa S, Bolibar BV, Roset M, Tomas R. Performance of an ambulatory casemix measurement system in primary care in Spain. European Journal of Public Health. 1999;9:27-35.

5. Reid RJ, MacWilliam L, Verhulst L, Roos N, Atkinson M. Performance of the ACG case-mix system in two Canadian provinces. Med Care. 2001;39(1):86-99.

6. Sicras-Mainar A. Validating the ACG Casemix System in a Spanish population setting: a crosssectional study. 2008.

7. Zielinski A, Kronogård M, Lenhoff H, Halling A. Validation of ACG Case-mix for equitable resource allocation in Swedish primary health care. BMC Public Health. 2009;9(347):1-8.

8. Haas LR, Takahashi PY, Shah ND, Stroebel RJ, Bernard ME, Finnie DM, et al. Risk-stratification methods for identifying patients for care coordination. The American journal of managed care. 2013;19(9):725-32.

9. Lemke KW, Weiner JP, Clark JMC. Development and Validation of a Model for Predicting Inpatient Hospitalization. Medical Care. 2012;50(2):131-9.

10. Bolaños-Carmona V, Ocana-Riola R, Prados-Torres A, Gutierrez-Cuadra. Variations in health services utilization by primary care patients. Health Services Management Research. 2002;15:116-25.

11. Wahls TL, Barnett MJ, Rosenthal GE. Predicting Resource Utilization in a Veterans Health Administration Primary Care Population Comparison of Methods Based on Diagnoses and Medications. Medical Care. 2004;42(2):123-8.

12. Pietz K, Ashton CM, McDonell M, Wray NP. Predicting Healthcare Costs in a Population of Veterans Affairs Beneficiaries Using Diagnosis-Based Risk Adjustment and Self-Reported Health Status. Medical Care. 2004;42(1027-1035).

13. Orueta J-F, Urraca J, Berraondo I, Darpon J, Aurrekoetxea J-J. Adjusted Clinical Groups (ACGs) explain the utilization of primary care in Spain based on information registered in the medical records: A cross-sectional study. Health Policy 2006;76:38-48.

14. Hanley GE, Morgan S, Reid RJ. Explaining prescription drug use and expenditures using the adjusted clinical groups case-mix system in the population of British Columbia, Canada. Medical Care. 2010;48(5):402-8.

15. Aguado A, Guinó E, Mukherjee B, Sicras A, Serrat J, Acedo M, et al. Variability in prescription drug expenditures explained by adjusted clinical groups (ACG) case-mix: A cross-sectional study of patient electronic records in primary care. BMC Health Services Research. 2008;8(53):1-11.

16. Chang H-Y, Weiner JP. An in-depth assessment of a diagnosis-based risk adjustment model based on national health insurance claims: the application of the Johns Hopkins Adjusted Clinical Group case-mix system in Taiwan. BMC Medicine. 2010;8(7):1-13.

17. Sicras-Mainar A. Adaptive capacity of the Adjusted Clinical Groups Case-Mix System to the cost of primary healthcare in Catalonia (Spain): a observational study. 2012.

18. Brilleman SL, Gravelle H, Hollinghurst S, Purdy S, Salisbury C, Windmeijer F. Keep it simple? Predicting primary health care costs with clinical morbidity measures. Journal of Health Economics. 2014;35(100):109-22.

19. Hughes JS, Averill RF, Eisenhandler J, Goldfield NI, Muldoon J, Neff JM, et al. Clinical Risk Groups (CRGs): a classification system for risk-adjusted capitation-based payment and health care management. Med Care. 2004;42(1):81-90.

20. García-Goñi M, Ibern P. Predictability of drug expenditures: an application using morbidity data. Health Economics 2006;17:119-26.

21. Vivas-Consuelo D, Usó-Talamantes R, Guadalajara-Olmeda N, Trillo-Mata J-L, Sancho-Mestre C, Buigues-Pastor L. Pharmaceutical cost management in an ambulatory setting using a risk adjustment tool. BMC Health Services Research. 2014;14(462):1-11.

22. Vivas-Consueloa D, Usó-Talamantesb R, Trillo-Matab JL, Caballer-Tarazonac M, Barrachina-Martíneza I, Buigues-Pastora L. Predictability of pharmaceutical spending in primary health services using Clinical Risk Groups. Health Policy 2014;116:188-95.

23. Fuller RL, Hughes JS, Goldfield NI. Adjusting Population Risk for Functional Health Status. Population health management. 2016.

24. Eissens van der Laan MR, van Offenbeek MAG, Broekhuis H, Slaets JPJ. A person-centred segmentation study in elderly care: Towards efficient demand-driven care. Social Science and Medicine. 2014;113:68-76.

25. Liu L-F. Utilization of health care services by elderly people with National Health Insurance in Taiwan: The heterogeneous health profile approach. 2012.

26. Lafortune L, Beland F, Bergman H, Ankri J. Health state profiles and service utilization in community-living elderly. Medical care. 2009;47(3):286-94.

27. Zhou YY, Wong W, Li H. Improving care for older adults: a model to segment the senior population. The Permanente journal. 2014;18(3):18-21.

28. Joynt KE, Figueroa JF, Beaulieu N, Wild RC, Orav EJ, Jha AK. Segmenting high-cost Medicare patients into potentially actionable cohorts. Healthc (Amst). 2017;5(1-2):62-7.

29. Low LL, Yan S, Kwan YH, Tan CS, Thumboo J. Assessing the validity of a data driven segmentation approach: A 4 year longitudinal study of healthcare utilization and mortality. PLoS One. 2018;13(4):e0195243.

30. Low LL, Kwan YH, Liu N, Jing X, Low ECT, Thumboo J. Evaluation of a practical expert defined approach to patient population segmentation: a case study in Singapore. BMC Health Serv Res. 2017;17(1):771.
